# Changji’an formula alleviates visceral hypersensitivity of a post-inflammatory IBS-D mouse model via NGF/TrkA signaling pathway

**DOI:** 10.1186/s12906-025-05095-3

**Published:** 2025-10-01

**Authors:** Wei Ke, Siyu Huang, He Zhu, Qinglong Tan, Huaiguo Li, Dongwen Liu, Fanghao Zheng, Shuncong Zhang, Kaijun Lei, Hongmei Tang

**Affiliations:** 1 The Eighth Clinical Medical College of Guangzhou University of Chinese Medicine, Foshan, Guangdong 528000 China; 2 Foshan Hospital of Traditional Chinese Medicine, No.6 Qinren Road, Foshan, Guangdong 528000 China; 3 Guangdong Provincial Key Laboratory for Research and Evaluation of Pharmaceutical Preparations, Guangdong Pharmaceutical University, Guangzhou, 510006 PR China; 4 The First Affiliated Hospital of Guangzhou University of Chinese Medicine, 16 Ji Chang Road, Guangzhou, Guangdong 510405 China; 5 Medical College of Shaoguan University, Shaoguan, Guangdong 512023 China

**Keywords:** Irritable bowel syndrome with predominant diarrhea, Changji'an formula, Visceral hypersensitivity, NGF/TrkA signal pathway

## Abstract

**Background:**

Changji’an Formula (CJAF) is an effective Chinese herbal prescription to treat irritable bowel syndrome with predominant diarrhea (IBS-D), which is derived from two famous classical prescription: Sijunzi decoction and Tong-Xie-Yao-Fang. However, the molecular mechanism has not been well defined. This study aims to illustrate the molecular mechanism of CJAF in the treatment of IBS-D.

**Methods:**

Chemical components of CJAF were determined by ultrahigh performance liquid chromatography-quadrupole/orbitrap high resolution mass spectrometry (UPLC-Q-Orbitrap HRMS) and further verified by reference standards. IBS-D model was induced in C57BL/6J mice by a single edema with colonic infusion of 0.1 mL trinitrobenzene sulfonic acid (TNBS, 50 mg/mL) combined with 7 days of restraint stress, 2 h/d. The treatment group was given rifaximin (100 mg/kg) and high, moderate and low doses of CJAF by gavage for 7 days, respectively (*n* = 7). After administration, the main symptoms of IBS-D were tested, and behavioral tests were conducted using sucrose preference test and open field test. The colonic tissues of mice were obtained. Gene and protein expression of mast cell tryptase, nerve growth factor (NGF), tyrosine kinase receptor A (TrkA), phosphorylated TrkA, growth associated protein 43(GAP43) and neuro-specific enolase (NSE) were determined by RT-PCR, western blot and immunohistochemistry.

**Results:**

36 compounds were identified by UPLC-Q-Orbitrap HRMS, and the determined components can be categorized into 7 chemical types, including 16 flavonoids, 7 triterpenoids, 4 alkaloids, 3 sesquiterpenoids, 2 monoterpene glycosides, 2 organic acids, 1 phenylpropanoids and 1 tannin. Animal experiment showed that the abdominal pain and diarrhea symptoms of IBS-D mice were alleviated by CJAF. The sucrose preference, total translocation distance and average velocity of movement in the open field test was upregulated. The mRNA and protein expression of mast cell marker tryptase, as well as NGF, phosphorylated TrkA, GAP43 and NSE in IBS-D mice colonic tissues were down-regulated by CJAF.

**Conclusions:**

CJAF could effectively alleviate abdominal pain and diarrhea symptoms of IBS-D by inhibiting the activation of colonic mast cells and the resultant activation of NGF/TrkA signal pathway. Therefore, CJAF affords a potential candidate for the treatment of IBS-D.

**Graphical Abstract:**

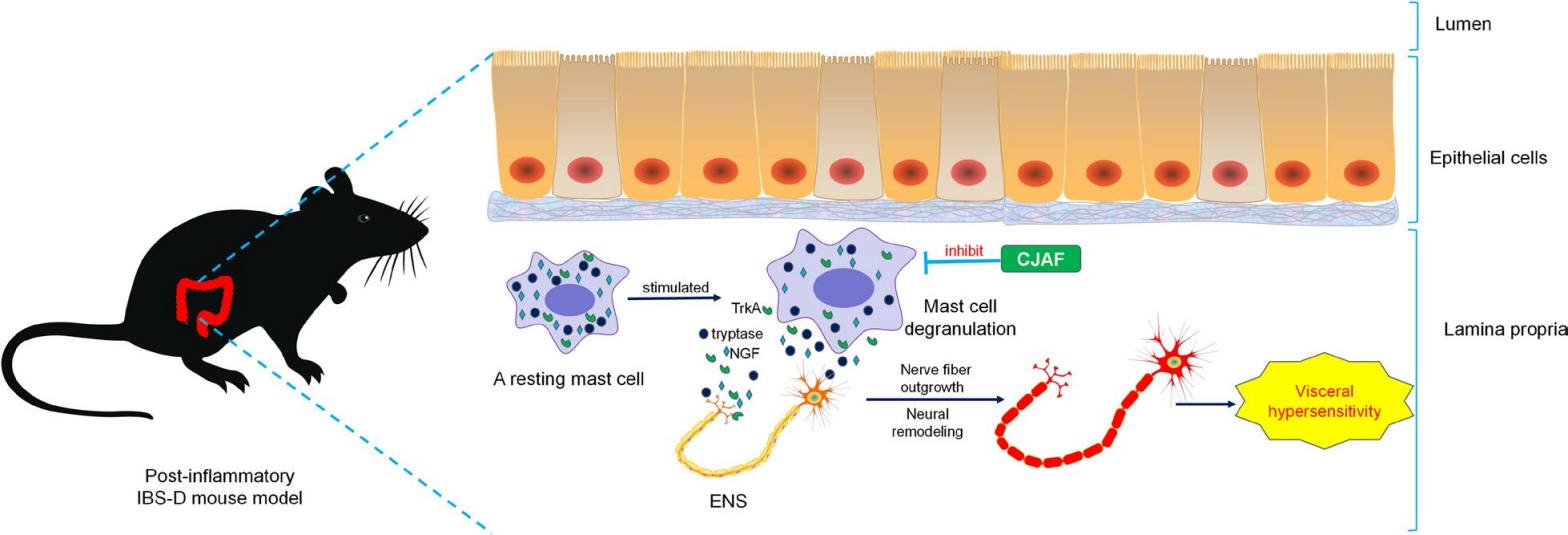

**Supplementary Information:**

The online version contains supplementary material available at 10.1186/s12906-025-05095-3.

## Introduction

Irritable bowel syndrome (IBS) is a common bowel disorder in which recurrent abdominal pain is associated with defecation or a change in bowel habits [1]. Based on Bristol stool form scale(BSFS) [2], IBS can be classified into four main types by Rome team [1]. Of the four subtypes, IBS-D is the most commonly-seen one, which poses a more severe influence on the life quality of patients [3, 4]. The pathogenesis of IBS-D is very complex, which is associated with genetic factors, visceral hypersensitivity, intestinal immune activation, abnormal gastrointestinal motility, enteric dysbacteriosis, involving nervous system, immune system and endocrine system [1]. Visceral hypersensitivity is regarded as an important pathological factor in IBS-D [5]. Visceral hypersensitivity refers to the increased sensitivity and reactivity of visceral tissues to mechanical and chemical stimuli. Enteric nervous system (ENS) is the biggest part of peripheral nervous system (PNS) in organism, which integrates between intestinal mucosal barrier and intestinal muscle layer. Sensory neurons located in ENS are key sensors in local neural circuits, where nociceptive neurons or nociceptors specifically transmit visceral pain and are therefore closely related to visceral hypersensitivity [6]. Previous studies have shown that the abnormal interaction between the immune activation in the gut and ENS can lead to intestinal neuropathy or structural remodeling, which is closely related to visceral hypersensitivity and ultimately lead to the pathogenesis of IBS [7 [8],. The activation of mast cells and the remodeling of nerve fibers play key roles in IBS peripheral sensitization. Researchers have shown that the activation of mast cells in the colon lead to the release of tryptase, histamine, nerve growth factor (NGF) and other mediators, resulting in outgrowth of intestinal mucosal nerve fiber, thus participating in IBS visceral hypersensitivity [9–11]. NGF is a member of the neurotrophic factor family, and the biological complex in which NGF functions is enabled by the binding of NGF to one of its two different neurotrophic receptors: TrkA and p75NTR, which normally preferentially binds to its dominant receptor: TrkA, to evoke nerve fiber growth and pain transmission [12]. Interestingly, the two receptors of NGF, TrkA and p75NTR, cooperate functionally to promote neuronal differentiation by upregulating the transcription of GAP43, which is an intrinsic determinant of neuronal development and plasticity and is closely involved in neuronal differentiation [13, 14]. However, the relationship between neuropathy of ENS and visceral hypersensitivity in IBS remains to be further clarified.

At present, the treatment strategy for IBS is complex and the effectiveness of drug therapy is quite limited [15]. Due to the large heterogeneity of IBS patients, a universal treatment strategy is often difficult to achieve. To make matters worse, other promising drugs have either been withdrawn from the market for various reasons or can only be used under more restricted conditions, making it urgent to develop effective treatments for IBS-D [15]. More and more studies have shown that TCM has satisfactory therapeutic effect on IBS-D in clinical practice [16, 17]. Chang ji’an Formula (CJAF) is the combination and transformation of two famous classical prescription: Sijunzi decoction and Tong-Xie-Yao-Fang, which have been widely used to treat IBS-like symptom diseases or IBS-D in clinic [17–19]. Moreover, our previous studies have shown that CJAF could regulate the expression of TNF-α, IL-10, IL-8, IL-1β and other inflammatory factors in IBS-D rat models, activate the JAK2/STAT3 pathway in the hippocampus of IBS-D rats, thus to regulate the immune function of IBS-D rat models, and reduce visceral hypersensitivity, playing a therapeutic role in the treatment of IBS-D [20–22]. However, whether the effective treatment of IBS-D by CJAF is related to the regulation of intestinal mast cell activation and the resultant improvement of immune function and ENS visceral sensation remains to be further clarified. Therefore, in the current study, the chemical components of CJAF were determined, and the mRNA and protein expression of mast cell tryptase, NGF, TrkA, GAP43 as well as NSE in colonic tissues of IBS-D mouse model administrated with CJAF was quantified.

## Materials and methods

### Regents

Formic acid, methanol, aqua pura used in UPLC-MS experiment were purchased from Thermo Fisher (Waltham, MA, USA). Trinitrobenzene sulfonic acid (TNBS) was obtained from Sigma-Aldrich (St. Louis, MO, USA, Lot#SLCD2161). The reverse transcription kit used for synthesizing cDNA was EVO M-MLV RT Premix for qPCR (Code NO.AG11706). The PCR kit used was the SYBR^®^ Green Premix Pro Taq HS qPCR kit (Code NO. AG11701), all purchased from Accurate Biotechnology Co., Ltd. (Changsha, China). TRIzol was obtained from Invitrogen Life Technologies (Invitrogen Life Technologies, Carlsbad, CA, USA). Western blot Stripping buffer is purchased from CW-BIO (lot number: 01427/12322, Jiangsu Province).

### Principal instruments

Ultrahigh performance liquid chromatography (UPLC I-Class, Waters, Milford, MA, USA) and Quadrupole orbital ion trap mass spectrometry (Q Exactive™, Thermo Scientific) was used in CJAF chemical composition analysis. Reversed phase column, ACQUITY UPLC HSS T3 (1.8 μm, 2.1 mm*100 mm), Waters was used. Fluorescent quantitative PCR instrument (US BioRad company, model CFX96 Touch) was used in PCR experiment. Low temperature centrifuge (eppendorf, model 5424R). Gel imager (US BioRad, model ChemiDoc MP), Digital slice scanner (Hungarian 3DHISTECH, model Pannoramic MIDI), and Transmission electron microscope (Japan HITACHI, model HT7800/HT7700) were common experimental equipment employed in the current study.

### Preparation of CJAF

The composition of CJAF is shown in Table 1, including the full scientific species (Latin binomial nomenclature) names and the exact mass of all herbs of CJAF.The preparation of CJAF extract was described previously [23]. In brief, the medicinal materials were weighed according to the prescribed dosage. Soak in 4 times the amount of water for 30 min, boil for 40 min, filter the Liquid medicine, add 3 times the amount of water to the residue, boil for another 30 min, filter out the liquid medicine, combine the liquid medicine together, filtrate and concentrate, and make them into lyophilized powder. Diluted the lyophilized powder into the corresponding concentration with pure water before use according to the yield rate of lyophilized powder.

**Table 1:** Medicinal materials information of changji’an formula (CJAF)

NO.	Chinese pinyin	English Name	Latin Name	Exact mass (g)
1	Baizhu	ATRACTYLODIS MACROCEPHALAE RHIZOMA	*Atractylodes macrocephala* Koidz.	30
2	Baishao	PAEONIAE RADIX ALBA	*Paeonia lactiflora* Pall.	15
3	Huangqi	ASTRAGALI RADIX	*Astragalus membranaceus* (Fisch.) Ege. var. *mongholicus* (Ege.) Hsiao	15
4	Fuling	PORIA	*Faria cocos*(Schw.)Wolf	20
5	Zhiqiao	AURANTII FRUCTUS	*Citrus aurantium* L.	10
6	Shiliupi	GRANATI PERICARPIUM	*Punica granatum* L.	30
7	Yanhusuo	CORYDALIS RHIZOMA	*Corydalis yanhusuo* W. T. Wang	10
8	Huanglian	COPTIDIS RHIZOMA	*Coptis chinensis* Franch.	5
9	Wumei	MUME FRUCTUS	*Prunus mume* (Sieb.) Sieb. et Zucc.	15
10	Fangfeng	SAPOSHNIKOVIAE RADIX	*Saposhnikovia divarzcata* (Turcz.) Schischk.	15
11	Chenpi	CITRI RETICULATAE PERICARPIUM	*Citrus reticulata* Blanco	5
12	Gancao	GLYCYRRHIZAE RADIX ET RHIZOMA	*Glycyrrhiza uralensis* Fisch.	6
13	Chaihu	BUPLEURI RADIX	*Bupleurum chinense* DC.	10

### Analysis of chemical constituents of CJAF by UPLC-Q-Orbitrap HRMS

The chemical constituents of CJAF was determined by UPLC-Q-Orbitrap HRMS. The separation was performed by UPLC I-Class Plus ultra-high performance liquid chromatography system (Waters Corporation, Milford, MA, USA). Chromatographic separations were performed on a Waters HSS T3 column (1.8 μm, 2.1 mm×100 mm). The column and autosampler temperature were maintained at 40 °C. A mobile phase consisting of 0.1% formic acid in water (A) and methanol (B) was applied with the optimized gradient program as follows:0–1 min, 2% (B); 1–41 min, 2%−100% (B); 41–50 min, 100% (B); 50–50.1 min, 100%−2% (B); 50.1–52.0 min, 2% (B). The flow rate was set at 0.3 mL/min. The injection volume was 10.0 µL. Mass spectrometry was performed using a quadrupole orbital ion trap mass spectrometer (Q Exactive™) (Thermo Fisher, Waltham, MA, USA) equipped with a thermoelectric spray ion source. The ion source voltages of positive and negative ions are 3.7 kV and 3.5 kV respectively. Capillary heating temperature is 320 ℃. Sheath gas pressure is 30 psi, auxiliary gas pressure is 10 psi. Solvent heating evaporation temperature is 300 ℃. Both sheath gas and auxiliary gas are nitrogen. The impact gas is nitrogen at 1.5 mTorr. The first level full scanning parameters are as follows: resolution 70 000, automatic gain control target 1 × 10^6^, maximum isolation time 50 ms, mass-charge ratio scanning range 100–1500. The mass axis of mass spectrum is calibrated by external standard method, and the mass error is 5 ppm. The compounds were identified using dd-MS2 scanning mode (data-dependent scanning mode) with the following parameters: resolution 17 500, automatic gain control target 1 × 10^5^, maximum isolation time 50 ms, scanning secondary fragments of up to 10 ions (dynamic exclusion), mass separation window 2, impact energy 30 V, and intensity Limit 1× 10^5^. Xcalibur 2.2SP1.48 software was used to control the liquid-mass system and collect data.

### Animals, IBS-D mouse model, grouping and drug administration

This study was approved by the Animal Ethics Committee of Guangzhou University of Chinese Medicine (Ethics approval number: 20220510014). Eight-week-old male C57BL/6J mice weighing 20–25 g was used in this study. Animals were handled in accordance with the regulations of Guangzhou University of Chinese Medicine and the ARRIVE Guidelines. All mice were free access to feed and water with a 12 h light-dark cycle (25 ± 1 °C). The mice were provided by the Laboratory Animal Center of Guangzhou University of Chinese Medicine. Animal experiments, grouping and drug administration was the same lot of our previous research [23]. In brief, after establishment of IBS-D mouse model, they were randomly divided into 6 groups(*n* = 7): NC, MC, rifaximin (Alanno, Italy; lot no. 22953) group, CJAF-H, CJAF-M, and CJAF-L. The reason to choose rifaximin as a positive drug was that clinical studies showed that rifaximin could effectively relieve general symptoms of IBS, such as abdominal pain, diarrheal and bloating [24, 25]. CJAF-H, CJAF-M and CJAF-L were equivalent to 2, 1, 1/2 times the clinical equivalent dose, respectively. After 7 days of continuous intragastric administration, relevant behavioral tests were carried out.

The mice were then anesthetized with pentobarbital sodium (50 mg/kg) via *i.p.* before the abdominal cavity was dissected by abdominal surgery. And the colonic tissues of the mice were cut out. After being washed with PBS, the colonic tissues were cut into serval segments, part of which was soaked in the paraformaldehyde fixing solution, and part was frozen in the refrigerator at −80 ℃ for further use. Then the mice were killed by cervical dislocation. Throughout the animal experiment, humane endpoints were confirmed if the weight of mice reduced more than 10%, or bloody stools developed for more than three consecutive days.

### Assessment of the main symptoms of IBS-D

The main symptoms of IBS-D, including mice diarrheal score, fecal water content, fecal pellet output under water 1 h of avoidance stress, and visceral sensitivity was assessed and reported in our previous report in this same lot of animal expreiment [23].

### Sucrose preference test

The detailed operation steps of sucrose preference test was conducted as described previously [26].

### Open field test

The procedure of open field test was conducted as previously described [27].

### Spleen coefficient and thymus coefficient

After administration of CJAF, the spleen and thymus of mice were obtained. After being washed with normal saline, the water was dried with dust-free filter paper, and the weight of spleen and thymus of mice was weighed and recorded for the calculation of organ index: spleen coefficient = weight of spleen(mg)/mice body weight(g), thymus coefficient = weight of thymus (mg)/mice body weight(g).

### mRNA expression of tryptase, NGF, TrkA, GAP43 and NSE by RT-qPCR

Total RNA was extracted from mouse colonic tissue and the integrity and purity of RNA was tested. RNA integrity and purity were considered acceptable only if OD260/OD280 ratio is between 1.8 and 2.1. After cDNA was synthesized by reverse transcription, primers of Tryptase, NGF, TrkA, GAP43 and NSE were added for Real-time PCR amplification. The PCR primers were synthesized by Shenggong Biotechnology (Shanghai) Co., Ltd. and the sequence of primers is shown in Table 2. Relative quantitation was performed by comparing CT values, with the formula F = 2^−△△CT^, where △△CT= (CT values of target genes in the test group - CT values of reference genes in the test group) - (CT values of target genes in the control group - CT values of reference genes in the control group). All reactions were performed in triplicate. mRNA expression was normalized to that of β-actin and compared with NC group.

**Table 2:** Primer sequences for RT-qPCR

Gene	Forward primer (5’ to 3’)	Reverse primer (3’ to 5’)
Tryptase Pro-NGF TrkA GAP43 NSE β-actin	CTGCGTGCCAATGACACCTAC GCGTTTTTGATCGGCGTACA CTTTGAGTACATGCGCCACG GAGGAGCCTAAACAAGCCGA TGCCAAAGGTCTTTTCCGGG GGGAAATCGTGCGTGAC	TACGGAGCTGTACTCTGACCTTG AGGGCTGTGTCAAGGGAATG CTAGCCACAGCCAGAAGCTG CTCATCCTGTCGGGCACTTT AACGCTGTTTGTCCCCATCC AGGCTGGAAAAGAGCCT

### Protein expression of tryptase, NGF, TrkA, GAP43 and NSE by Western blot

The total protein of mouse colonic tissue was extracted and the protein content was determined by BCA kit. After denaturation, the protein was wet transferred by polyacrylamide gel electrophoresis (SDS-PAGE electrophoresis) on a rotating machine at room temperature. After the film was transferred, it was blocked with 5% skim milk powder at room temperature (25 ℃) for 1 ~ 2 h, and then incubated in a refrigerator at 4 ℃ in a dark environment overnight (12 ~ 16 h) with the following antibody: Tryptase (1∶500, CAT. NO.: PA5-102552, Invitrogen, Waltham, MA, USA), anti-proNGF (1∶1000, CAT. NO.: ab52918, abcam, Cambridge, MA, USA), TrkA(1∶1000, CAT. NO.: ab76291, abcam, Cambridge, MA, USA), phosphorylated TrkA: Anti-TrkA (phospho Y496) + TrkB (phospho Y516) + TrkC (phospho Y516) (1∶1000, CAT. NO.: ab197071, abcam, Cambridge, MA, USA). GAP43(1∶1000, CAT. NO.: ab75810, abcam, Cambridge, MA, USA), NSE(1∶2000, CAT. NO.: ab79757, abcam, Cambridge, MA, USA), β-actin was used as internal reference (1∶2000, CAT. NO.: ab213262, abcam, Cambridge, MA, USA). After the primary antibody was recycled on the second day, PVDF membrane was washed with TBST and the secondary antibody (goat against rabbit IgG H&L (HRP) (ab205718, abcam, Cambridge, MA, USA)) was incubated at 25 ℃ for 1 h. After being washed with TBST, developer solution (Immobilon™ Western Chemiluminescent HRP Substrate, Millipore, WBKLS0050, USA) was added to the substrate and gel imager (Model ChemiDoc, MP BioRad, USA) was used to imaging. Finally, the protein imprinted bands were quantitatively analyzed by Image Lab software (Bio-Rad Laboratories, Hercules, CA, USA).

### Immunohistochemical analysis of tryptase, NGF, TrkA, GAP43 and NSE of mice colonic tissue

The detailed procedure was conducted as described previously [28]. Briefly, dewaxing paraffin sections with xylene and gradient concentration ethanol. Then proceeded with antigen repair procedure. Then the endogenous peroxidase was blocked by 3% hydrogen peroxide solution. Then the tissue was blocked with 3% BSA for 30 min. The blocking solution was gently cast off and the primary antibodies of Tryptase (dilution ratio 1:200), NGF (dilution ratio 1:250), TrkA (dilution ratio 1:1000), GAP43 (dilution ratio 1:500), NSE (dilution ratio 1:500) were added onto the sections. The primary antibodies used in immunochemistry were the same as used in Western blot assay. The slices were incubated with primary antibodies in a wet box at 4 ℃ overnight (8–16 h). Then the second antibody was added to cover tissue completely and incubated at room temperature for 50 min. Then the slices were dried and freshly prepared DAB color developing solution was added into the circle. After redyeing the nucleus, dehydration and neutral gum sealing were performed. Microscopic analysis: The sections were observed using the digital section scanning analysis system (3DHISTECH, Budapest, Hungary).

### Statistical analysis

Statistical analysis was performed using IBM SPSS v25.0 software (IBM Corp., Armonk, NY, USA). Shapiro-Wilk test was used to investigate the normality of data distribution. For comparison among multiple groups, parametric test is used if the data is normally distributed, otherwise non-parametric test is used. For parameter test, one-way analysis of variance (ANOVA) was used for the comparison among multiple groups, and the results were represented as mean ± standard deviation. As for *post hoc test*, if the variance is uniform, LSD-t is used; if the variance is not uniform, Dunnett’s T3 is used. Pairwise comparisons were used for post hoc tests. *P* < 0.05 was considered statistically significant. GraphPad Prism 8.0 software (GraphPad software Inc., San Diego, CA, USA) was used to plot, and error bars represented standard deviation (SD).

## Results

### Identification of the chemical constituents in CJAF

**Fig. 1:**
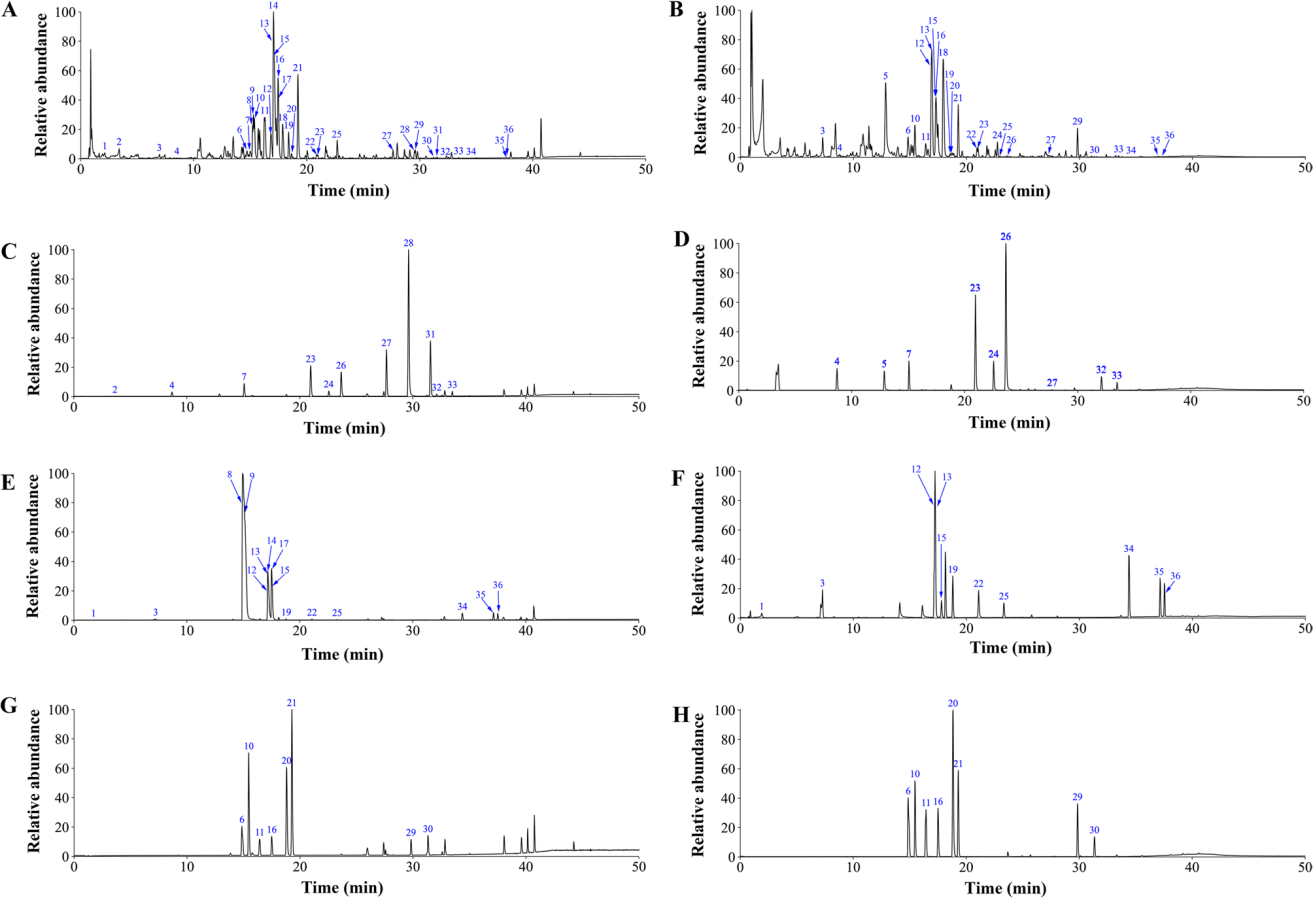
Chemical profiles of CJAF using UPLC-Q-Orbitrap HRMS. Representative ion chromatogram of CJAF in the (**A**) positive mode and in the (**B**) negative mode. 36 mixed reference standards based on UPLC-Q-Orbitrap HRMS chromatogram in (**C**), (**E**), (**G**)positive ion mode and in (**D**), (**F**), (**H**) negative ions mode

The chemical profiles of CJAF were investigated by UPLC-Q-Orbitrap HRMS analysis. Both positive and negative detection modes were used when collecting mass spectrometry data. Total ion chromatographs of CJAF decoction in positive mode (Fig. 1A) and negative mode (Fig. 1B) are demonstrated. Positive mode and negative mode of mixed solution of reference standards 1 (Fig. 1C-D), mixed solution of reference standards 2 (Fig. 1E-F), and mixed solution of reference standards 3 (Fig. 1G-H) were applied to validate the primary compounds of CJAF. Table 3 shows the results of UPLC-Q-Orbitrap HRMS analysis of CJAF and a total of 36 main components were identified. The determined components can be categorized into 7 chemical types, including 16 flavonoids, 7 triterpenoids, 4 alkaloids, 3 sesquiterpenoids, 2 monoterpene glycosides, 2 organic acids, 1 phenylpropanoids and 1 tannin.

**Table 3:** Identified ingredients of CJAF

NO.	Compounds	Classification	Molecular formula	Measured mass (m/z)	Theorical mass (m/z)	PubChem CID	RT(M+/M + H/M-H) min	Adduct ions (M + H/ M-H) mass (m/z)	Possible original resource
1	Citric Acid	organic acids	C_6_H_8_O_7_	192.0270	192.12	311	-/1.93/-	193.0348/191.0192	*Prunus mume* (Sieb.) Sieb. et Zucc.
2	Gallic acid	organic acids	C_7_H_6_O_5_	170.0215	170.12	370	-/3.42/-	171.0293/169.0137	*Paeonia lactiflora* Pall., *Punica granatum* L.
3	Neochlorogenic acid	Phenylpropanoids	C_16_H_18_O_9_	354.0951	354.31	5,280,633	-/7.05/7.19	355.1029/353.0873	*Prunus mume* (Sieb.) Sieb. et Zucc.
4	Catechin	flavonoids	C_15_H_14_O_6_	291.0868	290.27	9064	-/8.52/8.70	291.0868/289.0712	*Paeonia lactiflora* Pall.
5	Paeoniflorin	terpenoids	C_23_H_28_O_11_	480.1632	480.50	442,534	-/-/12.84	481.1710/479.1554	*Paeonia lactiflora* Pall.
6	Liquiritin	flavonoids	C_21_H_22_O_9_	418.1264	418.40	503,737	-/14.71/14.84	419.1342/417.1186	*Glycyrrhiza uralensis* Fisch.
7	Calycosin-7-O-beta-D-glucoside	flavonoids	C_22_H_22_O_10_	446.1213	446.40	5,318,267	-/14.97/-	447.1291/445.1135	*Astragalus membranaceus* (Fisch.) Ege. var. *mongholicus* (Ege.) Hsiao
8	Tetrahydropalmatine	alkaloids	C_21_H_26_NO_4_	356.1862	355.4	5417	15.22/-/-	357.1940/355.1784	*Corydalis yanhusuo* W. T. Wang
9	Coptisine	alkaloids	C_19_H_14_NO_4_^+^	320.0923	320.3	72,322	15.28/-/-	321.1001/319.0845	*Corydalis yanhusuo* W. T. Wang, *Coptis chinensis* Franch.
10	Prim-O-glucosylcimifugin	flavonoids	C_22_H_28_O_11_	468.1632	468.4	14,034,912	-/15.37/15.45	469.1710/467.1554	*Saposhnikovia divarzcata* (Turcz.) Schischk.
11	Narirutin	flavonoids	C_27_H_32_O_14_	580.1792	580.5	442,431	-/16.30/16.40	581.1870/579.1714	*Citrus reticulata* Blanco
12	Rutin	flavonoids	C_27_H_30_O_16_	610.1534	610.5	5,280,805	-/16.73/16.83	611.1612/609.1456	*Punica granatum* L., *Bupleurum chinense* DC.
13	Naringin	flavonoids	C_27_H_32_O_14_	580.1792	580.5	442,428	-/16.83/16.94	581.1870/579.1714	*Citrus aurantium* L.
14	Berberine	alkaloids	C_20_H_18_NO_4_^+^	336.1235	336.4	2353	17.06/-/-	337.1313/335.1157	*Coptis chinensis* Franch.
15	Ellagic acid	tannins	C_14_H_6_O_8_	302.0063	302.19	5,281,855	-/17.15/17.31	303.0141/300.9984	*Punica granatum* L.
16	Hesperidin	flavonoids	C_28_H_34_O_15_	610.1898	610.6	10,621	-/17.40/17.46	611.1976/609.1820	*Citrus reticulata* Blanco
17	Palmatine	alkaloids	C_21_H_22_NO_4_^+^	352.1548	352.5	19,009	17.47/-/-	353.1626/351.1470	*Corydalis yanhusuo* W. T. Wang, *Coptis chinensis* Franch.
18	Neohesperidin	flavonoids	C_28_H_34_O_15_	610.1898	610.6	442,439	-/17.88/17.94	611.1976/609.1819	*Citrus aurantium* L.
19	Quercitrin	flavonoids	C_21_H_20_O_11_	448.1006	448.4	5,280,459	-/18.46/18.57	449.1084/447.0928	*Punica granatum* L.
20	Liquiritigenin	flavonoids	C_15_H_12_O_4_	256.0736	256.25	114,829	-/18.70/18.80	257.0814/255.0658	*Glycyrrhiza uralensis* Fisch.
21	5-O-Methylvisammioside	flavonoids	C_22_H_28_O_10_	452.1682	452.5	21,670,038	-/19.22/19.27	453.1760/451.1604	*Saposhnikovia divarzcata* (Turcz.) Schischk.
22	Quercetin	flavonoids	C_15_H_10_O_7_	302.0427	302.23	5,280,343	-/20.65/20.76	303.0505/301.0349	*Punica granatum* L., *Bupleurum chinense* DC.
23	Calycosin	flavonoids	C_16_H_12_O_5_	284.0685	284.26	5,280,448	-/20.87/20.94	285.0763/283.0607	*Astragalus membranaceus* (Fisch.) Ege. var. *mongholicus* (Ege.) Hsiao
24	Benzoylpaeoniflorin	terpenoids	C_30_H_32_O_12_	584.1894	584.6	21,631,106	-/-/22.57	585.1972/583.1816	*Paeonia lactiflora* Pall.
25	Kaempferol	flavonoids	C_15_H_10_O_6_	286.0477	286.24	5,280,863	-/22.94/23.02	287.0555/285.0399	*Punica granatum* L.
26	Isoliquiritigenin	flavonoids	C_15_H_12_O_4_	256.0736	256.25	638,278	-/-/23.66	257.0814/255.0658	*Glycyrrhiza uralensis* Fisch.
27	Atractylenolide III	terpenoids	C_15_H_20_O_3_	248.1412	248.32	155,948	-/27.64/27.68	249.1490/247.1334	*Atractylodes macrocephala* Koidz.
28	Atractylenolide II	terpenoids	C_15_H_20_O_2_	232.1463	232.32	14,448,070	-/29.61/-	233.1541/231.1385	*Atractylodes macrocephala* Koidz.
29	Glycyrrhizic acid	triterpenoids	C_42_H_62_O_16_	822.4038	822.9	14,982	-/29.79/29.84	823.4116/821.3960	*Glycyrrhiza uralensis* Fisch.
30	Saikosaponin A	triterpenoid	C_42_H_68_O_13_	780.4660	781	167,928	-/31.30/31.34	781.4738/779.4582	*Bupleurum chinense* DC.
31	Atractylenolide I	terpenoids	C_15_H_18_O_2_	230.1307	230.3	5,321,018	-/31.54/-	231.1385/229.1229	*Atractylodes macrocephala* Koidz.
32	Astragaloside IV	triterpenoids	C_41_H_68_O_14_	784.4609	785	13,943,297	-/32.09/-	785.4687/783.4531	*Astragalus membranaceus* (Fisch.) Ege. var. *mongholicus* (Ege.) Hsiao
33	Saikosaponin D	triterpenoids	C_42_H_68_O_13_	780.4660	781	107,793	-/33.44/33.49	781.4738/779.4582	*Bupleurum chinense* DC.
34	Dehydrotumulosic acid	triterpenoids	C_31_H_48_O_4_	484.3552	484.7	15,225,964	-/34.36/34.39	485.3630/483.3474	*Faria cocos*(Schw.)Wolf
35	Dehydropachymic acid	triterpenoids	C_33_H_50_O_5_	526.3658	526.7	15,226,717	-/37.15/37.15	527.3736/525.3580	*Faria cocos*(Schw.)Wolf
36	Pachymic acid	triterpenoids	C_33_H_52_O_5_	528.3815	528.8	5,484,385	-/37.54/37.55	529.3893/527.3737	*Faria cocos*(Schw.)Wolf

*RT:* Retention time

### CJAF alleviated the main symptoms of IBS-D mice

The therapeutic effect of CJAF against IBS-D was described in the same lot of animal experiment previously [23]. The results demonstrated that CJAF significantly downregulated the diarrheal score, fecal water content, fecal pellet output under 1 h of WAS of IBS-D mice, and effectively alleviated visceral sensitivity of IBS-D mice. Therefore, CJAF relieved the main symptoms of IBS-D effectively.

### CJAF improved behavioral performance of IBS-D mice

**Fig. 2:**
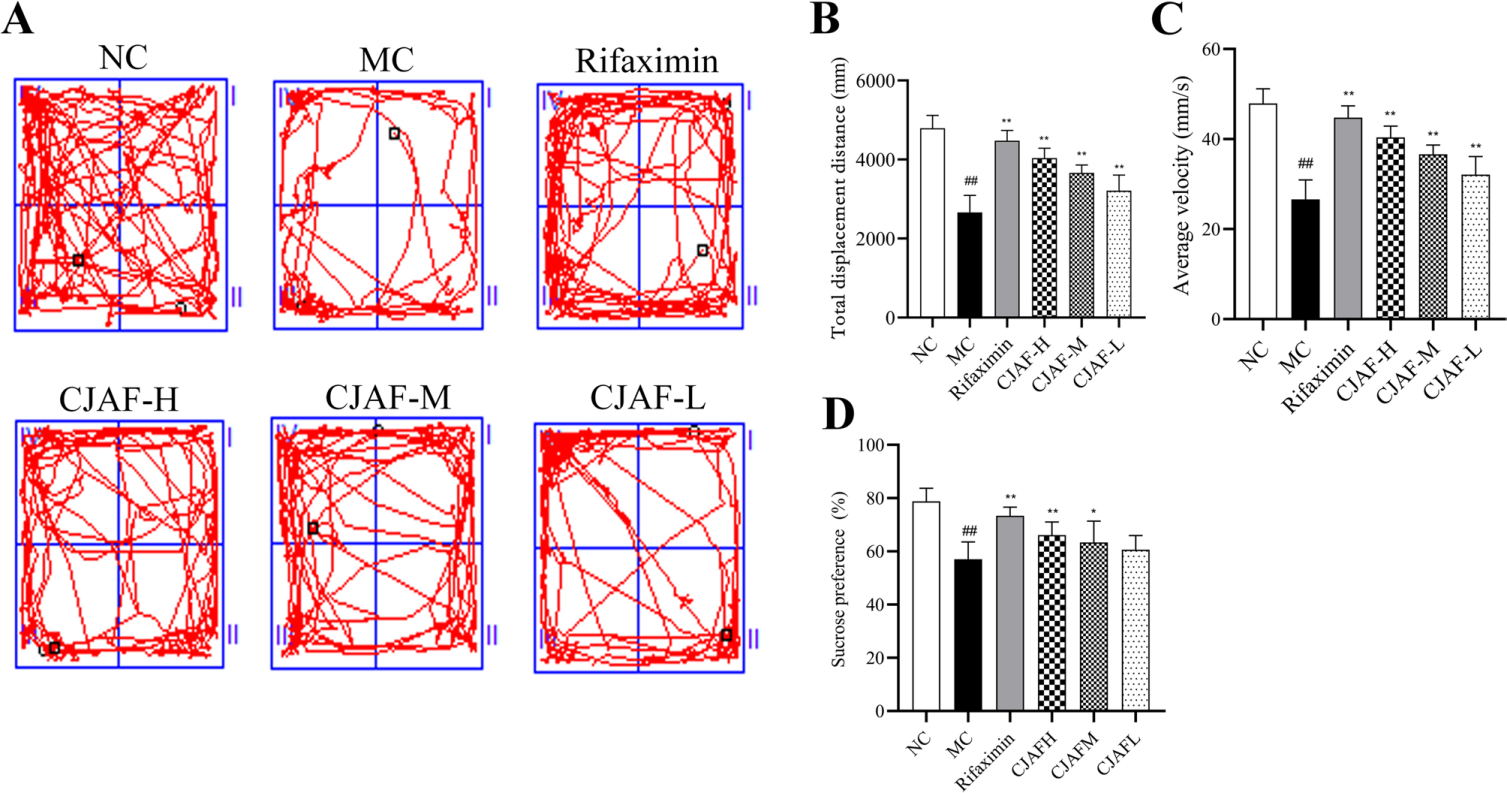
CJAF effectively improved the behavioral performance of IBS-D mice. (**A**) Ethology video analysis system demonstrated behavior trajectory diagram of mice; (**B**)total displacement distance and (**C**) average velocity of each group mice, (**D**) sucrose preference. The results are shown as. *n* = 7. (^##^*P* < 0.01 vs. NC; ^**^P < 0.01, ^*^P < 0.05 vs. MC)

The open field test is an experiment that explore spontaneous activity and exploratory behavior of animals to evaluate spontaneous activity, anxiety or depression of an animal [29]. Compare with normal mice, the spontaneous activity and exploration behavior of depressed mice will significantly decrease, and they keep far away from the open central area. Therefore, recording the behavior track and activity state of mice in the open field can make an objective evaluation of their mental state. Further, the sucrose preference test is a simple and easy behavioral test for measuring depressive or pleasurable behavior in animals. Depressed animals show loss of interest and decreased sucrose water preference [30]. As shown in Fig. 2A, IBS-D model mice were obviously afraid of the central open field area, and their behavior tracks were obviously concentrated on the four sides of the open field compared with the NC mice. The results of animal behavior video analysis system showed that compared with NC group, the total moving distance and average speed of mice in MC group decreased significantly (*P* < 0.01). After treatment with CJAF, the total moving distance and average speed of mice were significantly improved (*P* < 0.01) (Fig. 2B-C). The further experimental results of sucrose preference were consistent with the result of the open field test: compared with NC mice, the mice in MC group had a significant lower preference for sucrose water, and the mice recovered their preference for sucrose water after treatment with CJAF (*P* < 0.01 or *P* < 0.05) (Fig. 2D).

### CJAF downregulated mice spleen coefficient and thymus coefficient

**Fig. 3:**
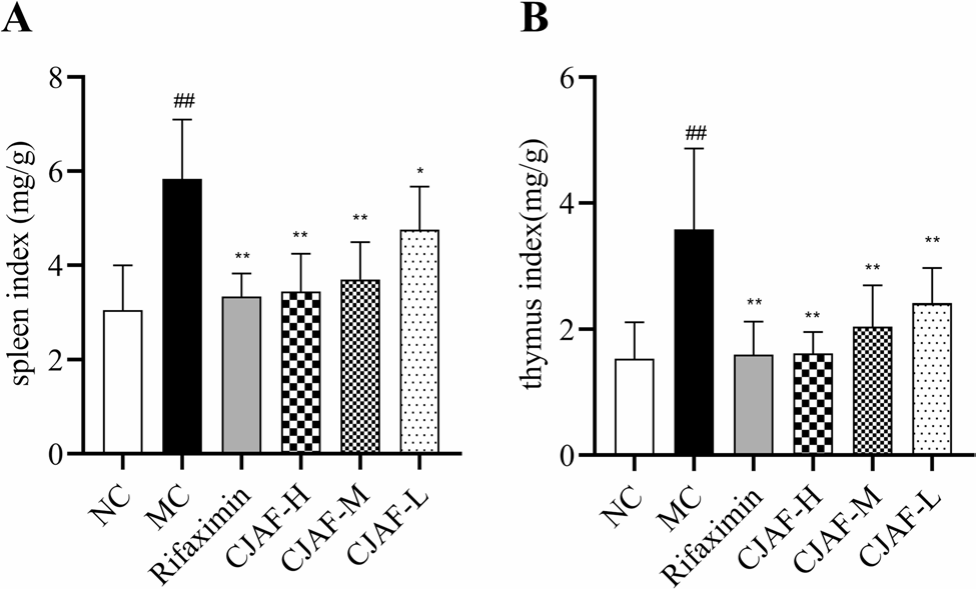
CJAF downregulated the spleen coefficient and thymus coefficient of IBS-D mice. Compared with NC, (**A**) the spleen index and (**B**) the thymus index in MC were significantly increased. After treatment with CJAF, (**A**) the spleen coefficient and (**B**) the thymus coefficient was downregulated dose-dependently. The results are shown as. *n* = 7. (^##^*P* < 0.01 vs. NC; ^**^P < 0.01, ^*^P < 0.05 vs. MC)

The spleen and thymus are important immune organs in organism, and the spleen coefficient and thymus coefficient are important indicators reflecting the immune function of the spleen and thymus, which play an important role in non-specific immunity of human body [31]. Many studies have shown that the body of patients with IBS-D is in a state of immune activation, and the overactivation of the immune system has significant influence on the pathogenesis of IBS-D [32–34]. Therefore, in this study, the spleen coefficient and thymus coefficient of mice were determined. The results showed that compared with NC, the spleen coefficient and thymus coefficient of IBS-D mice were significantly increased. However, after treatment with high, medium and low doses of CJAF, the spleen coefficient and thymus coefficient of mice were downregulated in different degrees(*P* < 0.01 or *P* < 0.05)༈Figure 3A-B༉.

### CJAF downregulated mRNA expression of tryptase, NGF, TrkA, GAP43 and NSE

**Fig. 4:**
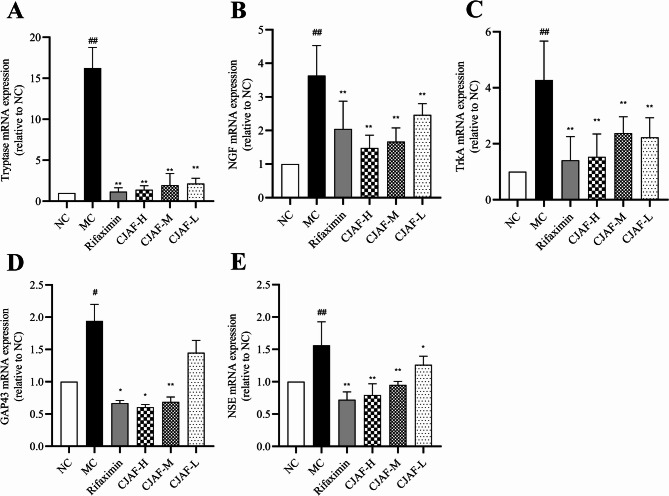
CJAF downregulated the mRNA expression of Tryptase, NGF, TrkA, GAP43 and NSE in mice colonic tissue. Compared with NC, the mRNA expression of Tryptase(**A**), NGF(**B**), TrkA(**C**), GAP43((**D**), NSE(**E**) in mouse colonic tissue was downregulated (*P* < 0.01 or *P* < 0.05). Changji’an Formula dose-dependently downregulated these targets (*P* < 0.01 or *P* < 0.05). Data are presented as, *n* = 4. (^##^*P* < 0.01, ^#^*P* < 0.05 vs. NC; ^**^P < 0.01, ^*^P < 0.05 vs. MC)

Compared with NC mice, the mRNA expression of Tryptase, NGF, TrkA, GAP43 and NSE in IBS-D mice colonic tissue were significantly upregulated (*P* < 0.01 or *P* < 0.05) (Fig. 4A-E). After treatment of high, moderate and low dose of CJAF, mRNA expression of Tryptase, NGF, TrkA, and NSE were significantly downregulated. The mRNA expression of GAP43 were significantly downregulated by high and moderate dose of CJAF (*P*_CJAF−H_ < 0.05 or *P*_CJAF−M_ < 0.01) (Fig. 4D), while low dose of CJAF showed no influence on mRNA expression of GAP43 (*P* > 0.05) (Fig. 4D).

### CJAF downregulated the protein expression of tryptase in mice colonic tissue

**Fig. 5:**
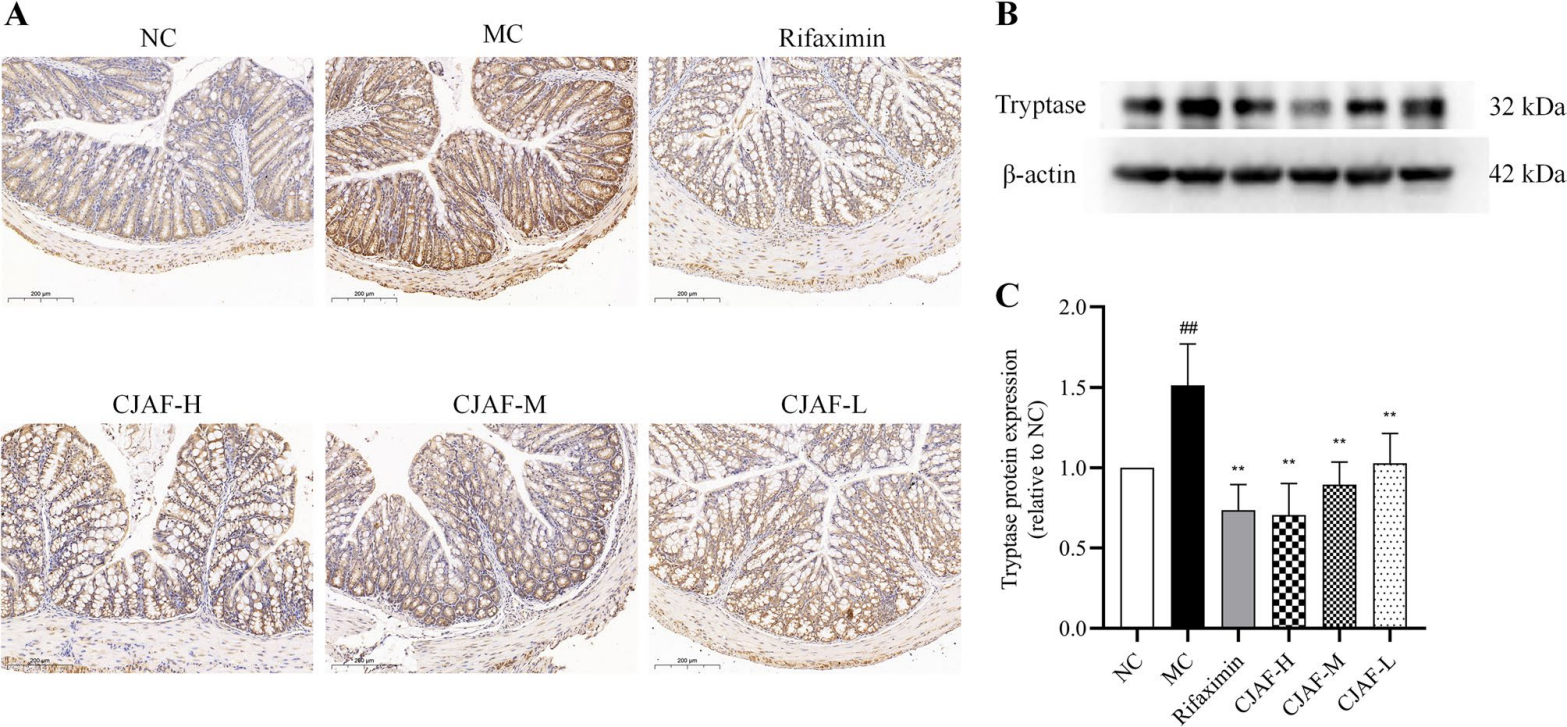
CJAF downregulated the protein expression of tryptase in mice colonic tissue. (**A**) Representative immunohistochemical staining of Tryptase in mice colonic tissue. (**B**) Representative western blot of Tryptase and (**C**) relative protein expression of Tryptase in mice colonic tissue. Data are presented as, *n* = 4. (^##^*P* < 0.01 vs. NC; ^**^P < 0.01 vs. MC). Full-length blots/gels are presented in Supplementary information

The activation of mast cells and the release of trypsin, NGF and other mediators are in the form of mast cell degranulation. These factors can induce intestinal mucosal immune activation and lead to visceral hypersensitivity [35, 36]. A number of studies have shown that the activation of colonic mucosal mast cells in patients with IBS-D and the release of tryptase and other mediators play an important role in the pathological mechanism of visceral hypersensitivity in IBS-D [35, 37]. Therefore, the expression of tryptase in colonic tissues of mice was detected in this study. As shown in Fig. 5A, compared with NC, the colonic tissue of IBS-D mice was significantly enhanced detected by immunohistochemical staining. Western blot was further used to quantitatively determine tryptase expression in mouse colonic tissue, and the results showed that IBS-D model induction significantly up-regulated the expression of tryptase in mouse colonic tissue(*P* < 0.01), while CJAF could downregulate the expression of tryptase in a dose-dependent manner(*P* < 0.01) (Fig. 5B-C).

### CJAF downregulated the protein expression of NGF in mice colonic tissue

Previous studies have shown that mast cells can synthesize, store and release NGF [38]. Researchers reported that mast cell infiltration increased in the colonic mucosal of patients with IBS and neurotrophic factors released by mast cells, mainly including NGF, can induce the change of colonic mucosal neuroplasticity and nerve fiber sprouting, resulting in visceral hypersensitivity in IBS [11]. Studies have shown that the increase of colonic mucosal NGF expression in patients with IBS-D is closely related to visceral hypersensitivity and impaired intestinal epithelial barrier function. In view of this, we examined the changes of NGF protein expression in mouse colonic tissue in the current study.

**Fig. 6:**
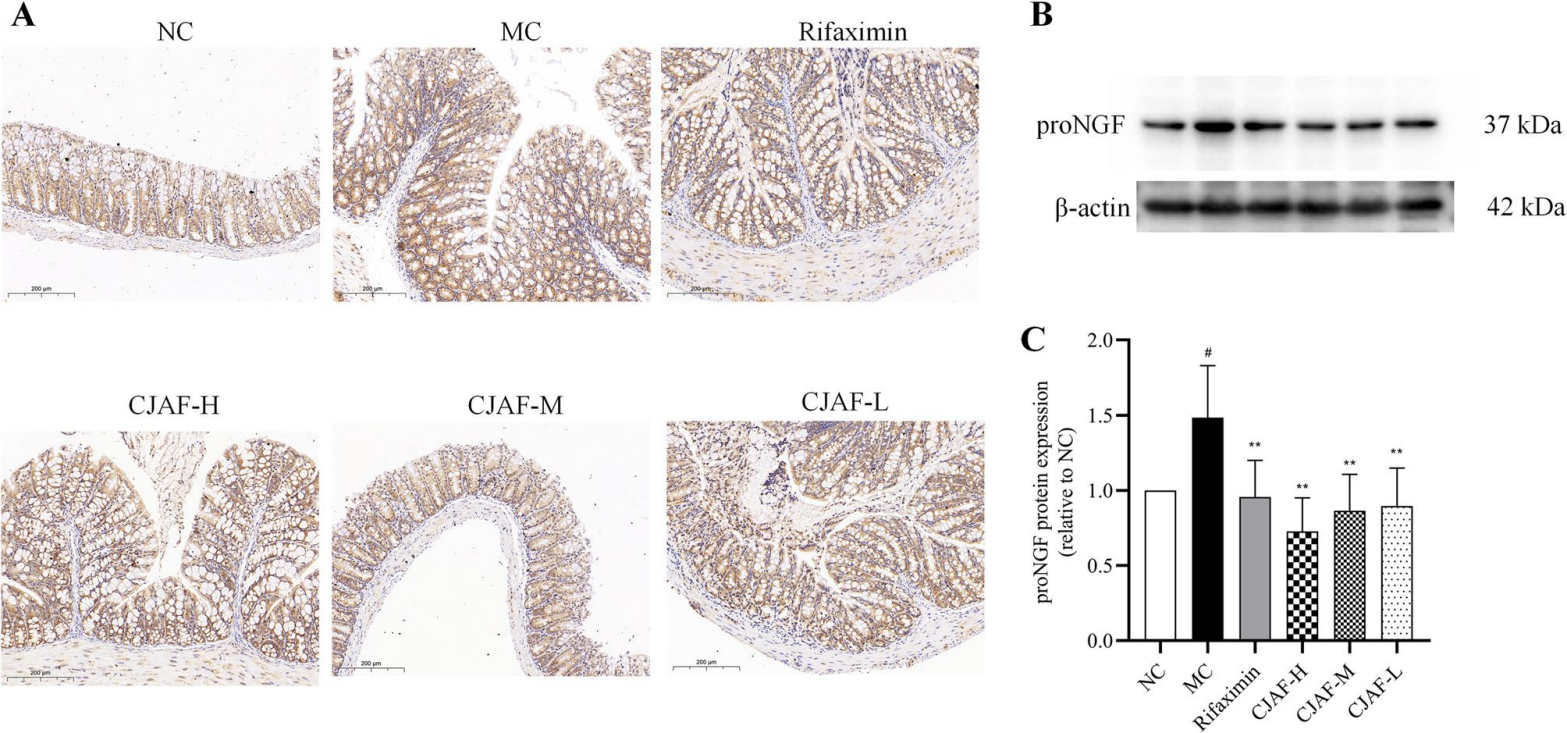
CJAF downregulated the protein expression of NGF in mice colonic tissue. (**A**) Representative immunohistochemical staining of NGF in mice colonic tissue. (**B**) Representative western blot of NGF and (**C**) relative protein expression of NGF in mice colonic tissue. Data are presented as, *n* = 4. (^#^*P* < 0.05 vs. NC; ^**^P < 0.01 vs. MC). Full-length blots/gels are presented in Supplementary information

As shown in Fig. 6A, the NGF immunohistochemical staining of colonic tissue of IBS-D model mice was significantly enhanced, and the intensity of immunohistochemical staining of NGF in mice colonic tissue was reduced to varying degrees after treatment with CJAF. Further quantitative results of western blot showed that the expression of NGF in colonic tissue of mice in MC group was significantly higher than that of NC group (Fig. 6B-C) (*P* < 0.05), while the expression of NGF protein was decreased by different doses of CJAF(*P* < 0.01).

### CJAF downregulated the protein expression of phosphorylated TrkA in mice colonic tissue

**Fig. 7:**
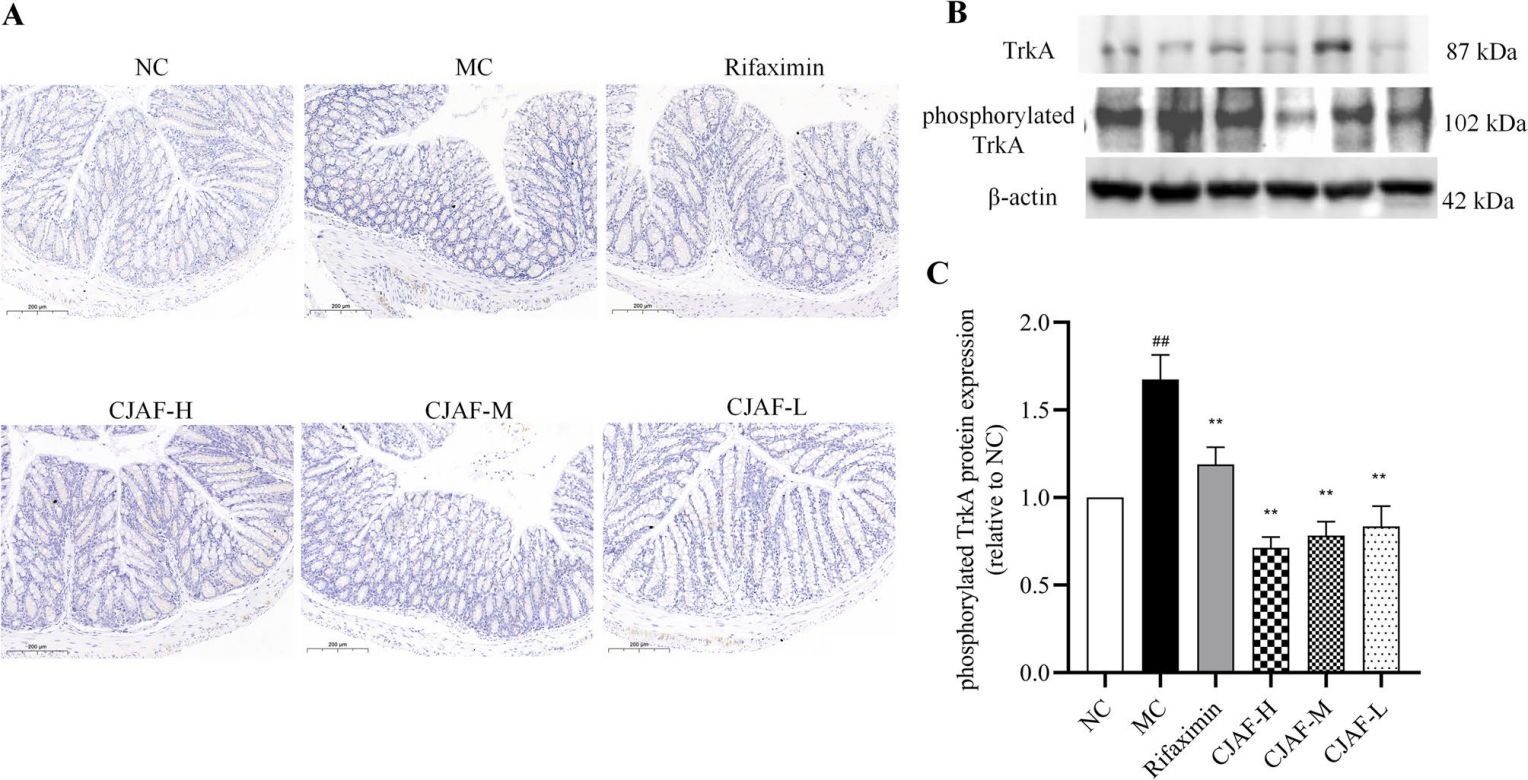
CJAF downregulated the protein expression of phosphorylated TrkA in mice colonic tissue. (**A**) Representative immunohistochemical staining of TrkA in mice colonic tissue. (**B**) Representative western blot of TrkA and phosphorylated TrkA; (**C**) relative protein expression of phosphorylated TrkA in mice colonic tissue. Data are presented as, *n* = 4. (^##^*P* < 0.01 vs. NC; ^**^P < 0.01 vs. MC). Full-length blots/gels are presented in Supplementary information

Previous studies showed that compared to NGF’s low-affinity receptor p75NTR, TrkA is NGF’s dominant receptor, and when NGF binds to TrkA, it causes nerve fiber outgrowth and pain transmission [13]. In addition, mast cells express both NGF and TrkA under inflammatory response conditions, the consequences of which participates in the production of inflammation-related visceral hypersensitivity [39]. Therefore, we determined the expression of TrkA in the colonic tissue of mice in the present study. Different from the results reported in literature that the expression of TrkA in the colonic mucosa of IBS was increased [11], the current study did not detect increased expression of TrkA protein in IBS-D model mice. Neither a change of TrkA protein expression was detected after the intervention of CJAF (Fig. 7A-C). However, the expression of phosphorylated TrkA in colonic tissue of mice in MC group was significantly higher than that of NC group (Fig. 7B-C) (*P* < 0.05), while the expression of phosphorylated TrkA protein was decreased after different doses of CJAF treatment (*P* < 0.01).

### CJAF downregulated the protein expression of GAP43 in mice colonic tissue

**Fig. 8:**
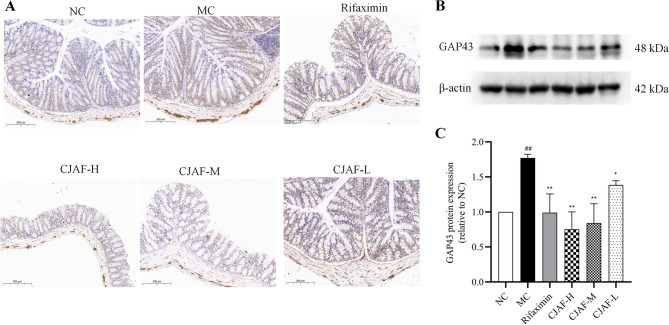
CJAF downregulated the protein expression of GAP43 in mice colonic tissue. (**A**) Representative immunohistochemical staining of GAP43 in mice colonic tissue. (**B**) Representative western blot of GAP43 and (**C**) relative protein expression of GAP43 in mice colonic tissue. Data are presented as, *n* = 4. (^##^*P* < 0.01 vs. NC; ^**^P < 0.01, ^*^P < 0.05 vs. MC). Full-length blots/gels are presented in Supplementary information

GAP-43 is a membrane-binding acidic protein that is an intrinsic presynaptic determinant of neurite growth and plasticity formation [40]. Previous studies have shown that the density of GAP43-positive fiber in colonic mucosal in IBS patients is higher than that of in healthy subjects [11]. Therefore, we compared the protein expression of GAP43 after CJAF treatment. As shown in Fig. 8A, the immunohistochemical staining of GAP43 in colonic tissue of mice in IBS-D model group was significantly enhanced compared with that of NC group, and the distribution of GAP43 was specific, mainly distributed in the submucosa of intestinal cavity and intestinal muscle layer. Further analysis by western blot showed that the expression of GAP43 protein in the colonic tissue of MC mice was significantly up-regulated compared with that of NC (*P* < 0.05), and the expression of GAP43 protein was down-regulated in a dose-dependent manner by CJAF, compared with that of MC (*P* < 0.01 or *P* < 0.05) (Fig. 8B-C).

### CJAF downregulated the protein expression of NSE in mice colonic tissue

**Fig. 9:**
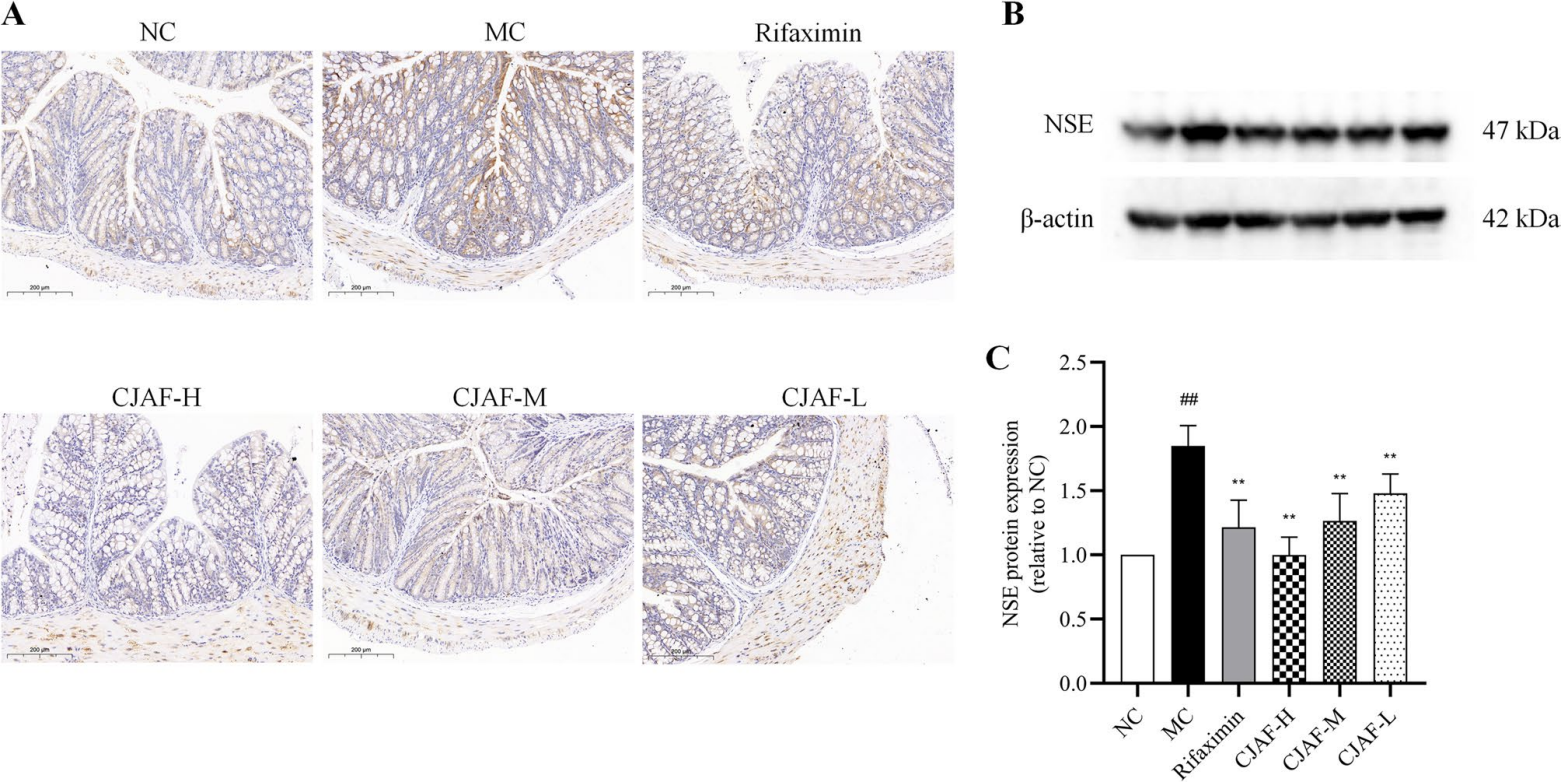
CJAF downregulated the protein expression of NSE in mice colonic tissue.(**A**) Representative immunohistochemical staining of NSE in mice colonic tissue. (**B**) Representative western blot of NSE and (**C**) relative protein expression of NSE in mice colonic tissue. Data are presented as, *n* = 4. (^##^*P* < 0.01 vs. NC; ^**^P < 0.01 vs. MC). Full-length blots/gels are presented in Supplementary information

Neuron-specific enolase (NSE) is an isoenzyme of the enolase glycolytic enzyme and a specific marker of ENS [41, 42]. Previous studies have shown that the number of NSE-positive nerve fibers in colonic mucosa in patients with IBS is significantly higher than that in healthy subjects [11]. Therefore, we determined the protein expression of NSE in the colonic tissue of mice in the current study. The results showed that IBS-D model induced significantly upregulated the expression of NSE in mouse colonic tissues, while high, medium and low doses of CJAF could down-regulate NSE expression level (*P* < 0.01) (Fig. 9A-C).

## Discussion

The aim of the current study was to investigate the mechanism of CJAF in the treatment of a post-inflammatory IBS-D mouse model. The results showed that the post-inflammatory IBS-D mouse model was successfully induced by TNBS enema combined with restraint stress. The symptoms of IBS-D were mainly manifested as increased visceral sensitivity, diarrhea, and behavioral changes such as reduced total moving distance in the open field, decreased average velocity, and decreased sucrose water preference. CJAF treatment could effectively inhibit the expression of tryptase in the colonic tissue of IBS-D mice and downregulate the expressions of NGF, phosphorylated TrkA, GAP43, NSE, and reduce the spleen coefficient and thymus coefficient of IBS-D mice, thereby inhibiting the immune activation and alleviating the pathological symptoms of abdominal pain and diarrhea of IBS-D mice.

Most animal models of IBS-D mimic the clinical symptoms of IBS-D by colorectal stimulation with chemical reagents or by stress, or the combination of the two, which induces diarrhea symptom and persistent visceral hypersensitivity of experimental animals. The most commonly used chemical enemas are acetic acid [43, 44] or ethanol solutions of TNBS [45, 46]. And the manners inducing stress include maternal separation (MS) [47], restraint stress [48] and water avoidance stress [49]. The combination of chemical stimulation with stress were also reported [50, 51]. In our preliminary experiment, by measuring relevant indicators such as AWR, fecal water content, and TNF-α expression in the colon, we found that TNBS enema combined with restraint stress was more effective than any single method and could establish a more stable IBS-D model. Therefore, in the current study, IBS-D model was constructed by TNBS enema combined with restraint stress.

Previous studies have shown that the colonic mucosal mast cell infiltration increases in patients with IBS, and the severity of IBS symptoms is closely related to activated mast cells [52, 53]. Activated mast cells play a key role in mediating neuro-immune interactions in IBS, because the bioactive mediators released by mast cells may lead to alterations in neuroplasticity that play an important role in abnormal movement, secretion, and pain perception in clinically commonly-seen gastrointestinal diseases such as IBS [54]. Mast cells could release a variety of mediators once activated in the form of degranulation, the most typical of which is tryptase, a signature protein of mast cells. These active mediators can stimulate visceral pain sensory nerves, and are closely related to the perception of abdominal pain in IBS [9, 55]. In this study, the gene and protein expression of tryptase in the colon of IBS-D were upregulated, and the spleen coefficient and thymus coefficient were also significantly increased, indicating that the mast cells were activated and the immune state was activated in the IBS-D model. However, after the treatment of CJAF, the expression of tryptase in mice colonic tissue was inhibited, and the spleen index as well as the thymus index of mice were effectively downregulated, which indicated that CJAF could effectively inhibit the excessive immune activation of IBS-D mice.

Antidepressants or psychotherapy are beneficial for functional disorders such as IBS-D, not only because of their effects on the central nervous system, but also because of their effects on the peripheral nervous system, such as pain perception, visceral hypersensitivity, and gastrointestinal motility [56, 57]. Indeed, psychological factors have long been thought to be important factors in the pathogenesis of IBS-D, and patients with IBS-D are often accompanied by psychological disorders, and the latter, such as depression or anxiety, can also increase the risk of developing IBS-D [58–60]. Therefore, this study tested the behavior of mice through the open field test and sugar water preference experiment to investigate the effect of CJAF on behavioral performance of IBS-D mice. The results showed that the total moving distance and average velocity of IBS-D model mice were significantly reduced, the movement tracks mainly concentrated around the open field, not in the central area, and IBS-D mice demonstrated a reduced preference for sugar solution water, which indicated a state of depression. CJAF treatment significantly improved the activity state of mice, increased the total moving distance and average speed of mice in the open field, and restored the preference of mice to sucrose water. The results of mouse behavioral tests in this study further support the view that psychological factors play a role in the pathological mechanism of IBS-D.

Previous studies have shown that neuro-immune factors play an important role in the pathogenesis of IBS-D, and this neuro-immune interaction is closely related to pathological changes in ENS. Intestinal mast cells and its released bioactive mediators are closely involved in neuro-immune interactions. These mediators include common neurotrophic factors, such as NGF, could cause visceral hypersensitivity in IBS [61, 62]. Previous studies have shown that colonic mucosal mast cell infiltration increases in patients with IBS, and NGF, the mediator released by which, can induce neuroplasticity changes of ENS and neuron outgrowth [11]. The results of the current study are consistent with that reported: the expression of NGF in the colonic tissue of mice in IBS-D model group is significantly upregulated, and CJAF could dose-dependently downregulate the expression of NGF. NSE is a general marker of intestinal neurons, and previous studies have shown that the expression of NSE-positive nerve fibers in colonic mucosa of patients with IBS is increased [11]. Therefore, the expression of TrkA, GAP43 and NSE in mouse colonic tissues were also determined in this study. However, this study failed to detect changes in the expression of TrkA protein in the colonic tissue of IBS-D model mice, but the expression of phosphorylated TrkA in colonic tissue of mice in MC group was significantly higher than that of NC group, while the expression of phosphorylated TrkA protein was decreased after different doses of CJAF treatment. This is of great significance: The phosphorylated TrkA (phosphorylated TrkA, p-TrkA) has protein kinase activity, and p-TrkA can activate a series of signal transduction systems to exert biological effects, such as to promote cell proliferation and survival [63, 64], neuron growth and neuronal neurite outgrowth [65–67] and be involved with in both health and disease states [68].

Therefore, the mechanism of the effective treatment of IBS-D by CJAF may be due to its inhibiting the activation of mast cells and down-regulate the expression of NGF, GAP43 and NSE, so as to effectively attenuate hyperalgesia. Several bioactive chemical components of CJAF may exert this regulatory effect on the activation of mast cells and thus affect the immune state in IBS-D. For example, paeoniflorin is a monoterpene glycoside in *Paeonia lactiflora* Pall., which was detected in CJAF by UPLC-Q-Orbitrap HRMS, demonstrating inhibitory effect on mast cell degranulation [69, 70]. Berberine, an isoquinine alkaloid from *Coptis chinensis* Franch in CJAF, could significantly alleviate chronic WAS-induced visceral hypersensitivity and activation of colonic mast cells in rat models of visceral hypersensitivity [71]. Rutin is a widely-found chemical compound in medicinal plants, such as *Punica granatum* L. and *Bupleurum chinense* DC. in CJAF, which was proved to alleviate inflammatory pain by inhibiting the activity of P2 × 7 receptor in mast cells [72]. As activation of mast cells in the colonic mucosa of IBS patients induces neuroplastic changes and neuronal sprouting via the release of NGF [11], chemical ingredients in CJAF that inhibit the activation of mast cells could, therefore, exert therapeutic effect on IBS-D.

The novelty of our research lies in the fact that we have demonstrated that CJAF not only locally regulates the NGF/TrkA pathway in the intestine to alleviate visceral hypersensitivity in IBS-D, but also modulates the behavior of IBS-D model mice, such as the total distance moved and average speed in the open field test and sucrose preference. This not only further indicates the significance of the brain-gut axis abnormality in the pathological mechanism of IBS-D, but also demonstrates that CJAF, a traditional Chinese medicine compound, exerts a multi-faceted and comprehensive therapeutic effect on IBS-D, including the alleviation of local visceral hypersensitivity in the intestine and possibly the regulation of the central perception.

## Conclusion

CJAF could not only inhibit the activation of colonic mast cells in IBS-D, down-regulate the expression of tryptase, reduce the expression of NGF, phosphorylated TrkA, GAP43 and NSE, thereby regulating abnormal ENS perception and reducing visceral hypersensitivity, but also could effectively improve the behavioral performance of IBS-D mice, thus exerted therapeutic effect in the treatment of IBS-D.

## Supplementary Information

Supplementary Material 1.

## Data Availability

The data used to support the findings of this study are available from the corresponding author upon reasonable request.

## Abbreviations

IBS-D: Irritable Bowel Syndrome with Predominant Diarrhea
CJAF: Changji'an Formula
UPLC-Q-Orbitrap HRMS: Ultrahigh Performance Liquid Chromatography-quadrupole/orbitrap High Resolution Mass Spectrometry
TNBS: Trinitrobenzene Sulfonic Acid
AWR: Abdominal Withdrawal Reflex
NGF: Nerve Growth Factor
TrkA: Tyrosine Kinase Receptor A
GAP43: Growth Associated Protein 43
NSE: Neuro-specific Enolase
ENS: Enteric Nervous System
