# Supplementary material for: Changji’an formula alleviates visceral hypersensitivity of a post-inflammatory IBS-D mouse model via NGF/TrkA signaling pathway
Source: BMC Complement Med Ther. 2025 Oct 1;25:348. doi: 10.1186/s12906-025-05095-3 (PMC12487093; doi:10.1186/s12906-025-05095-3)
Supplement: Supplementary file 1 — Supplementary Material 1. [file 12906_2025_5095_MOESM1_ESM.pptx]

## Slide 1
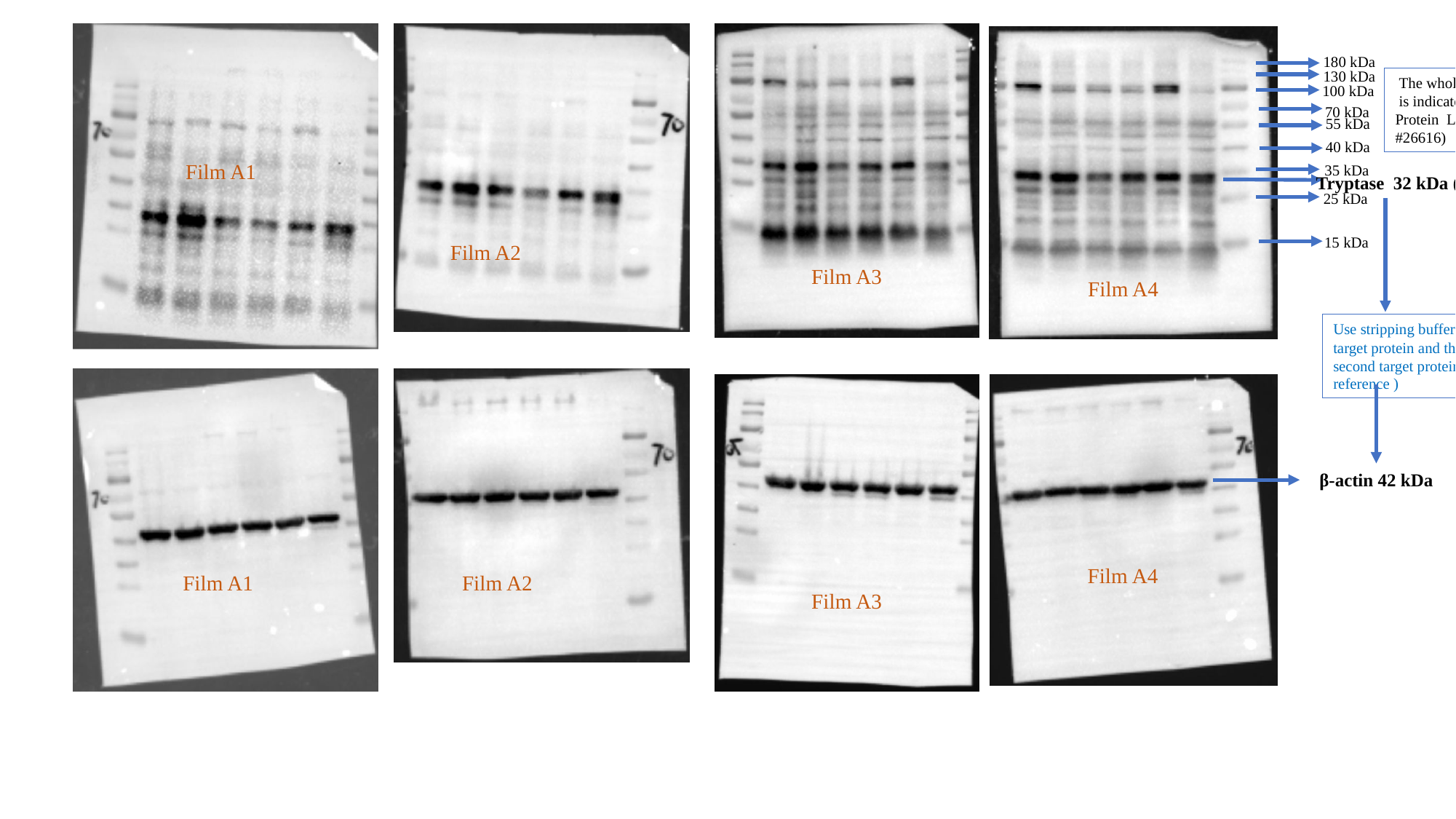

180 kDa
130 kDa
 The whole PVDF film
 is indicated by PageRuler Prestained Protein Ladder (Thermo Scientific, #26616)
100 kDa
70 kDa
55 kDa
40 kDa
Film A1
35 kDa
Tryptase 32 kDa (n=4)
25 kDa
15 kDa
Film A2
Film A3
Film A4
Use stripping buffer to strip out the first target protein and then incubate with the second target protein(internal reference )
β-actin 42 kDa
Film A4
Film A2
Film A1
Film A3

## Slide 2
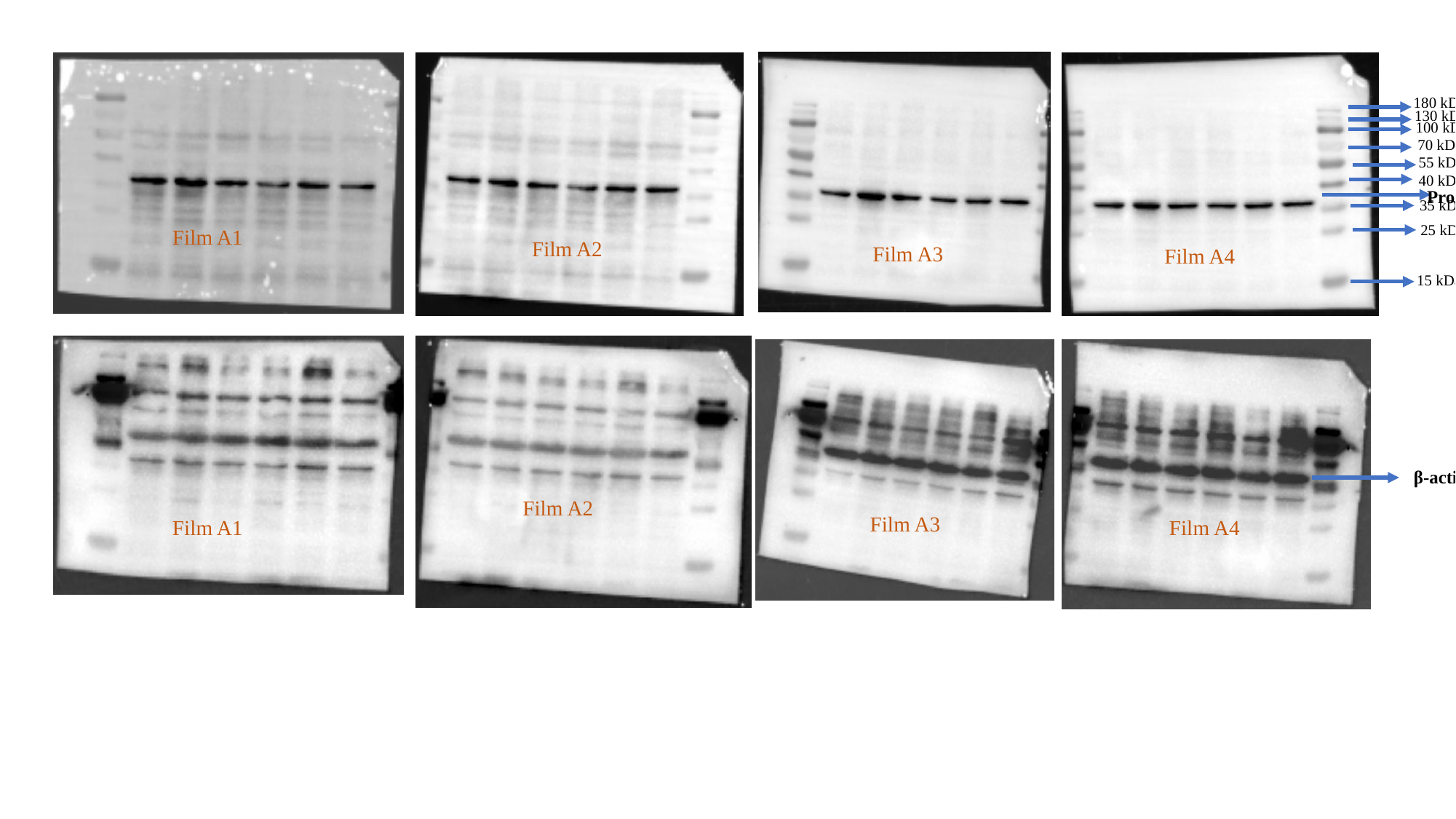

The whole PVDF film
 is indicated by PageRuler Prestained Protein Ladder (Thermo Scientific, #26616)
180 kDa
130 kDa
100 kDa
70 kDa
55 kDa
40 kDa
Pro-NGF 37 kDa (n=4)
35 kDa
25 kDa
Film A1
Film A2
Film A3
Film A4
15 kDa
Use stripping buffer to strip out the first target protein and then incubate with the second target protein(internal reference )
β-actin 42 kDa
Film A2
Film A3
Film A1
Film A4

## Slide 3
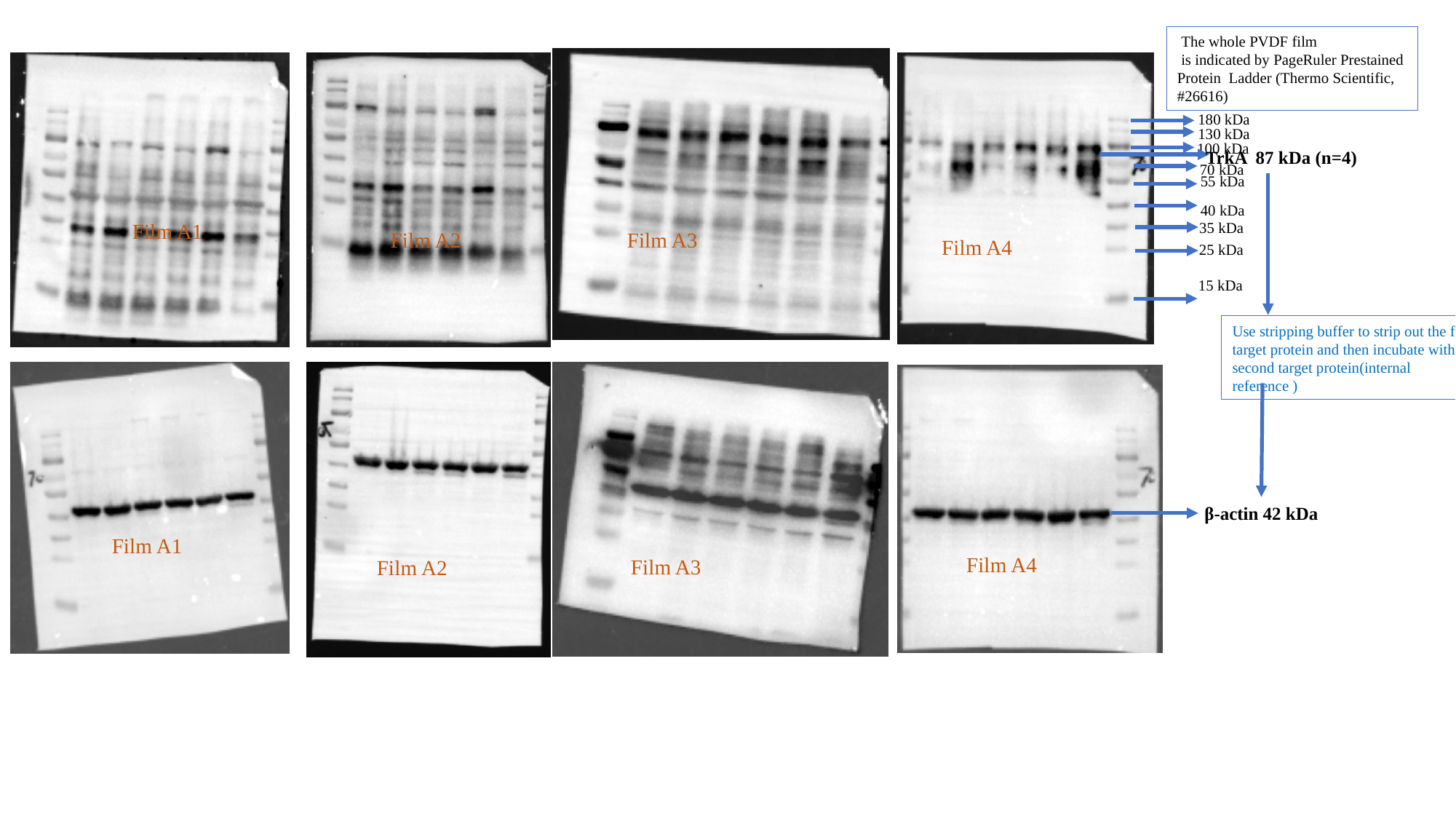

The whole PVDF film
 is indicated by PageRuler Prestained Protein Ladder (Thermo Scientific, #26616)
180 kDa
130 kDa
100 kDa
TrkA 87 kDa (n=4)
70 kDa
55 kDa
40 kDa
Film A1
35 kDa
Film A3
Film A2
Film A4
25 kDa
15 kDa
Use stripping buffer to strip out the first target protein and then incubate with the second target protein(internal reference )
β-actin 42 kDa
Film A1
Film A4
Film A3
Film A2

## Slide 4
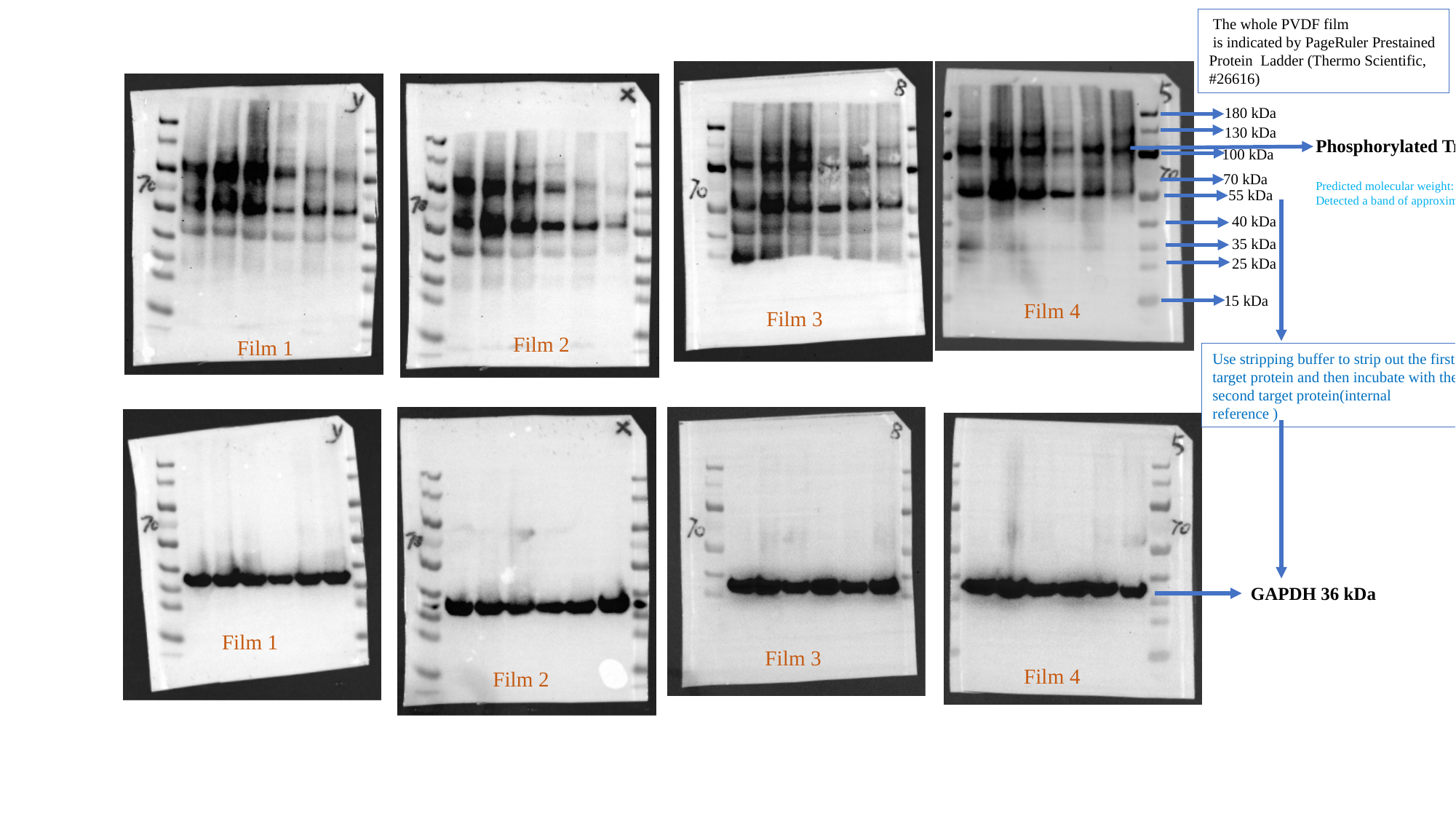

The whole PVDF film
 is indicated by PageRuler Prestained Protein Ladder (Thermo Scientific, #26616)
180 kDa
130 kDa
Phosphorylated TrkA(102kDa,n=4)
Predicted molecular weight: 92 kDa,
Detected a band of approximately 102 kDa (n=4)
100 kDa
70 kDa
55 kDa
40 kDa
35 kDa
25 kDa
15 kDa
Film 4
Film 3
Film 2
Film 1
Use stripping buffer to strip out the first target protein and then incubate with the second target protein(internal reference )
GAPDH 36 kDa
Film 1
Film 3
Film 4
Film 2

## Slide 5
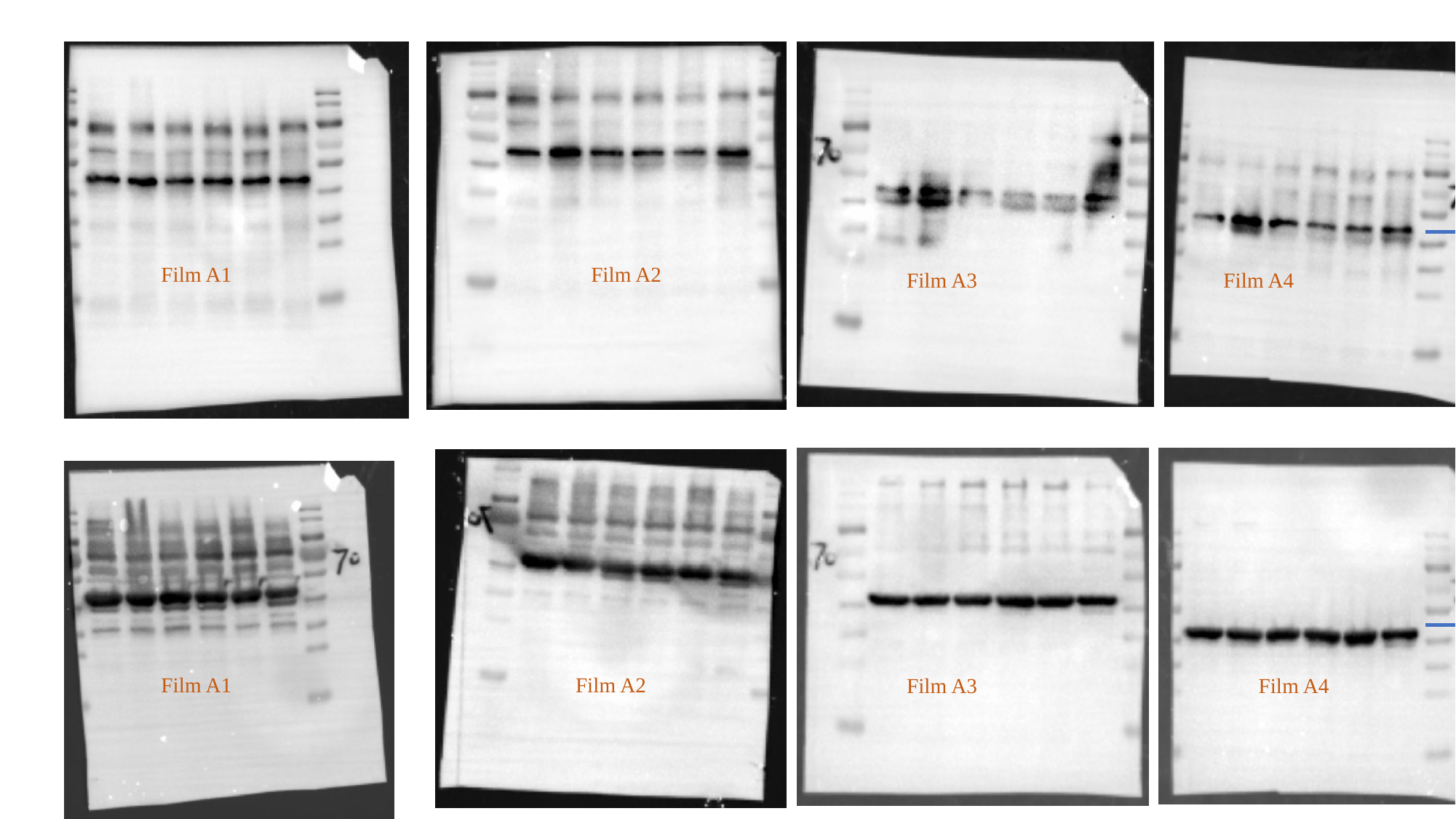

180 kDa
 The whole PVDF film
 is indicated by PageRuler Prestained Protein Ladder (Thermo Scientific, #26616)
130 kDa
100 kDa
70 kDa
55 kDa
GAP43 48 kDa (n=4)
40 kDa
Film A2
35 kDa
Film A1
Film A4
Film A3
25 kDa
Use stripping buffer to strip out the first target protein and then incubate with the second target protein(internal reference )
15 kDa
β-actin 42 kDa
Film A2
Film A1
Film A3
Film A4

## Slide 6
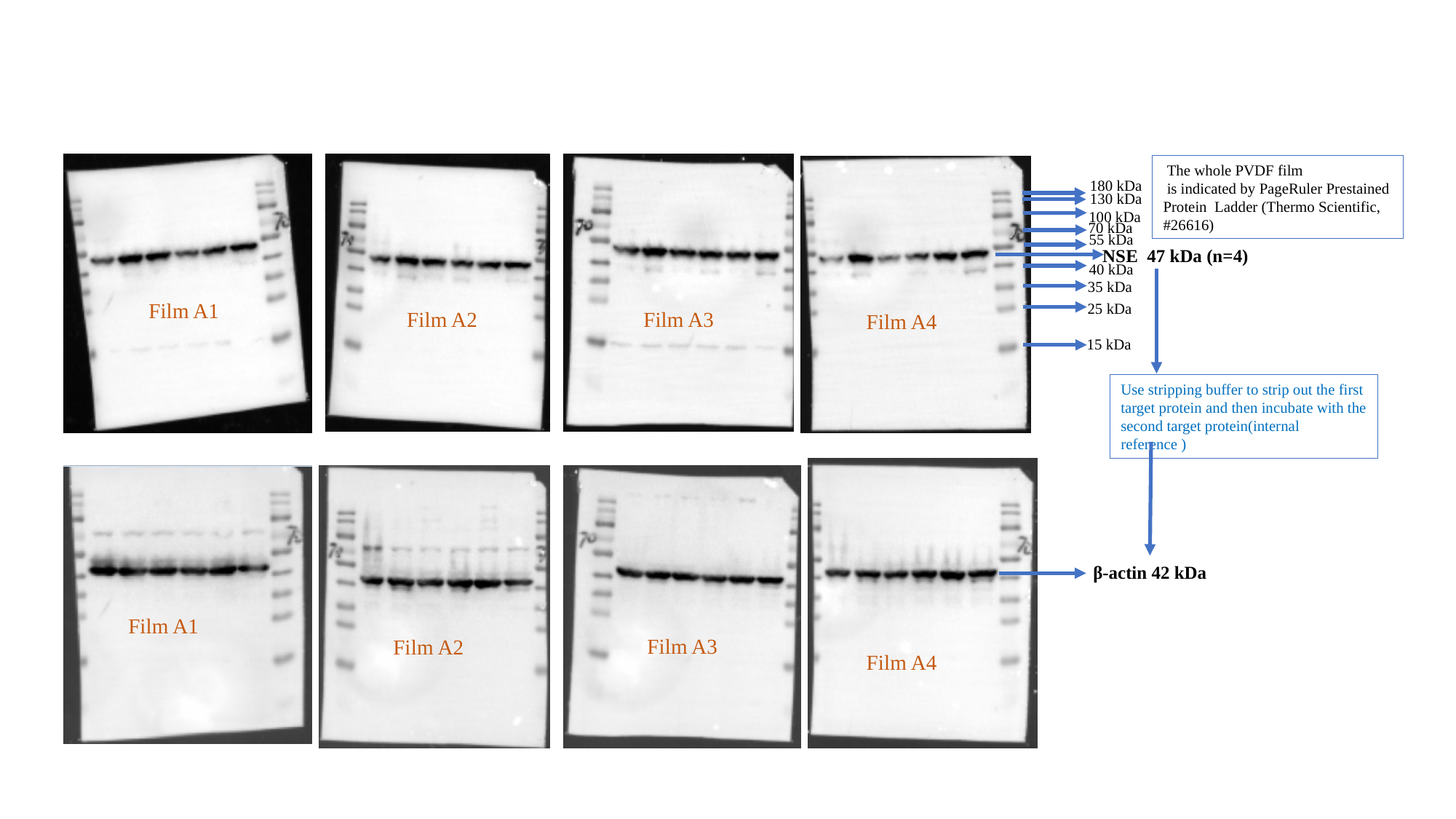

The whole PVDF film
 is indicated by PageRuler Prestained Protein Ladder (Thermo Scientific, #26616)
180 kDa
130 kDa
100 kDa
70 kDa
55 kDa
NSE 47 kDa (n=4)
40 kDa
35 kDa
Film A1
25 kDa
Film A3
Film A2
Film A4
15 kDa
Use stripping buffer to strip out the first target protein and then incubate with the second target protein(internal reference )
β-actin 42 kDa
Film A1
Film A3
Film A2
Film A4
